# Systematic review of economic evaluations of triage tests for women with atypical squamous cells of undetermined significance (ASC-US) or low-grade squamous intraepithelial lesions (LSIL)

**DOI:** 10.1017/S0266462324000540

**Published:** 2024-11-18

**Authors:** Isandra Meirelles, Márcia Pinto, Leticia Barros, Fabio Russomano

**Affiliations:** Instituto Nacional de Saúde da Mulher, da Criança e do Adolescente Fernandes Figueira, Fundação Oswaldo Cruz, Rio de Janeiro, Brasil

**Keywords:** Systematic Review, Cost-Effectiveness Analysis, Uterine Cervical Neoplasms, Squamous Intraepithelial Lesions of the Cervix

## Abstract

**Objectives:**

To synthesize the results of cost-effectiveness studies of different triage tests in comparison to repeat cytology for women with atypical squamous cells of undetermined significance (ASC-US) or low-grade squamous intraepithelial lesions (LSIL) results.

**Methods:**

Electronic databases (Medline/PubMed, Lilacs, Embase, The Cochrane Library, Scopus, Web of Science, Scielo, The NHS Economic Evaluation Database, Econlit, and CEA Registry) were searched for cost-effectiveness or cost-utility publications. Per the Preferred Reporting Items for Systematic Reviews and Meta-Analyses (PRISMA) guidelines, two independent reviewers selected eligible publications based on the selection criteria and performed data extraction. Methodological quality was assessed using the Quality of Health Economic Studies tool.

**Results:**

Five cost-effectiveness analyses were included comparing HPV testing, immediate colposcopy, and liquid-based cytology with HPV testing reflex to repeat cytology. The main outcome adopted was cervical intraepithelial neoplasia level 2 or higher (CIN2+) cases detected. In pairwise comparisons, HPV testing was more frequently observed as the most cost-effective strategy. Incremental cost-effectiveness ratios were very sensitive to costs of test kit variation and accuracy estimates with some sensitivity analysis scenarios showing immediate colposcopy more cost-effective than HPV testing depending on the tests’ unitary costs and effectiveness.

**Conclusions:**

This systematic review of economic evidence corroborates clinical evidence showing cytology is the least effective, although less costly, triage strategy. Cytology-based triage programs need to be updated to offer timely treatment to women diagnosed with ASC-US/LSIL and better resource allocation.

## Introduction

The burden of disease attributed to cervical cancer is the leading cause of disability-adjusted life years lost in women in forty-nine countries and is also associated with premature death in twenty-three countries (1). Global estimates reveal that in 2020, cervical cancer was the eighth most incident cancer in individuals above 15 years old, with approximately 600,000 new cases, and was the fourth most incidence among women, with an estimated 19.3 cases per 100,000 (2).

The World Health Organization (WHO) launched the global strategy for the Elimination of Cervical Cancer in 2020, aiming to have 70 percent of women worldwide regularly screened with high-performance tests, in addition to other goals related to vaccination, diagnosis, and treatment of identified precursor lesions. The HPV-DNA test is recommended as the preferred screening method, replacing cytology and visual inspection with acetic acid, which are the most used tests in developing countries (3).

Cytology is also the most used test in the triage phase due to its high specificity, although it has limited sensitivity and variability in interpreting results, besides false-negative results caused by sampling error or detection error (4).

Another well-known challenge faced in the follow-up phase is the low adherence to screening programs. The rate of inadequate follow-up ranges from 28.3 to 75 percent in cytology-based triage (5).

No international consensus exists about the better triage strategy, especially in developing countries, where women with atypical squamous cells of undetermined significance (ASC-US) or low-grade intraepithelial lesions (LSIL) undergo repeat cytology testing between 6 and 12 months depending on age and type of lesion (6,7). The main clinical guidelines recommend HPV tests alone or co-testing with cytology for these cases (8).

The use of a triage test aims to identify women with a higher probability of being carriers of precursor lesions or cancer and reduces the health system burden. Molecular tests with high specificity avoid unnecessary referrals for colposcopy or treatment (9).

For triaging women with ASC-US/LSIL cytology, tests based on HPV-DNA and RNA detection, detection of oncoproteins such as p16 and Ki-67, or DNA methylation can be used (10). Colposcopy can also be used as a triage test, but it implies higher costs and over-referral of healthy women to biopsy or treatment, misplacing attention from the most probable carriers of precursor lesions or cancer.

In Brazil, in the last 5 years, the proportion of exams with ASC-US/LSIL results represents 2.2 percent of the total number of exams performed and 69.5 percent of altered results (11), which would represent referring more than twice as many women per year for colposcopy, compromising the offer of exams for other women with cytological abnormalities more likely to represent cancer or its precursor lesions.

Given the diversity of technologies for reducing morbidity and mortality attributed to cervical cancer, high-quality evidence-based decisions are necessary for more efficient and effective screening programs, minimizing costs and increasing benefits to women’s health and quality of life. Health economic evaluations support such decisions and assist in defining public policies for the control and prevention of cervical cancer, providing economic evidence of triage strategies that should be analyzed together with clinical evidence (12–16).

Decision models are mathematical structures that reproduce the clinical and economic outcomes of alternative strategies in a simplified way, based on information collected from the context being evaluated. When developing models according to the natural history of the disease and diagnostic and treatment guidelines, in addition to the evaluation period or time horizon and the types of events that will be modeled, it is also important to consider the perspective of the analysis, which dictates the types of costs that will be reproduced in the model. Among the existing perspectives, those of the healthcare system, society, and patient are highlighted (17).

The results of economic evaluations in healthcare can be indicated by a ratio of health outcomes in natural units, such as life years gained or number of lives saved, and monetary units, producing the incremental cost-effectiveness ratio (ICER) (17). An alternative for better comparison of healthcare interventions in different contexts is the adoption of quality-adjusted life years (QALY) (18).

This parameter is strongly related to health utilities measured by several methods being the most successful approach to apply psychometric instruments in a questionnaire format, such as EQ-5D, to a representative sample of people to assess their preferences about life duration with life quality (18).

The quantification of utility, denoted by a numerical scale ranging from 0 to 1 where “0” signifies the most adverse health condition and “1” denotes optimal health, is a customary practice in health-related assessments. These utility values are conventionally employed in the computation of QALYs through their multiplication by the duration of time spent in specific health states. This systematic approach allows for a standardized representation of health outcomes, facilitating a comprehensive evaluation of the overall impact on quality of life (19). In a comprehensive search of the literature, four systematic reviews of cost-effectiveness evaluations of cervical cancer screening strategies were identified (20–23), but none evaluated cost-effectiveness studies of triage tests for women with ASC-US or LSIL cytology results.

In this study, we conducted a systematic review of primary studies that sought to estimate the cost-effectiveness of triage alternatives after primary cytology with ASC-US or LSIL results in different population contexts.

## Methods

This systematic review was conducted in accordance with the Preferred Reporting Items for Systematic Reviews and Meta-Analyses (PRISMA) (24). The protocol was registered in the International Prospective Register of Systematic Reviews (PROSPERO), with the registration code CRD42021237140, before the start of this review.

Eligible studies were complete economic analysis (cost-effectiveness and cost-utility) with any analytic time horizon, according to the following question:

- Population: Adult women who started screening and presented ASC-US/LSIL in primary cytology.
- Intervention: Repeat cytology for referral to colposcopy or treatment.
- Comparator: Any other triage strategy
- Outcome: ICER

Cost analysis and economic evaluations without a comparative result of costs and benefits expected were excluded.

Articles published up to January 2024 were searched in the following databases: Medline/Pubmed, Lilacs, Embase, and The NHS Economic Evaluation Database. In addition to the databases, a hand-search in the references list of the included studies was also conducted. The sample search strings can be found in Supplementary Material.

The search strategy was constructed using controlled vocabulary terms (Health Descriptors—DECS/Medical Subject Headings—MESH), synonyms, truncation, and variations in the search fields of the databases combined with boolean operators to prevent the loss of any relevant publications. No date or language restrictions were applied.

Using the Rayyan® software, two reviewers (IM and LB) screened the titles and abstracts of studies identified in the electronic search. Full-text articles whose titles and abstracts were considered relevant by at least one reviewer were retrieved. Disagreements were solved by a third reviewer (MP).

The same two independent reviewers performed data extraction filling out a standardized data collection form on Microsoft Word^®^ and assessed the methodological quality of included studies using the Quality of Health Economic Studies (QHES) tool. It corresponds to a set of criteria with weights assigned according to their relevance in economic evaluation studies. The quality score is determined by summing up the points assigned to each “yes” response for answered questions (25).

The QHES employs a scoring system ranging from 0 (indicating the lowest quality) to 100 (reflecting the highest quality). This continuous scale can be dichotomized, categorizing studies into high-quality (75–100 points) and not high-quality (0–74 points) based on their respective scores (26).

The profile of studies and their characteristics were presented in tables, allowing for a comparison of economic and health outcomes.

To enable comparison of the ICER reported in each study, values were monetarily updated to 2024 and converted to U.S. dollars (US$). The costs were updated by inflating the local currency using local inflation rates between the date of publication and January 2024, and then exchanging to US$.

## Results

### Study selection and characteristics

**Figure 1.**
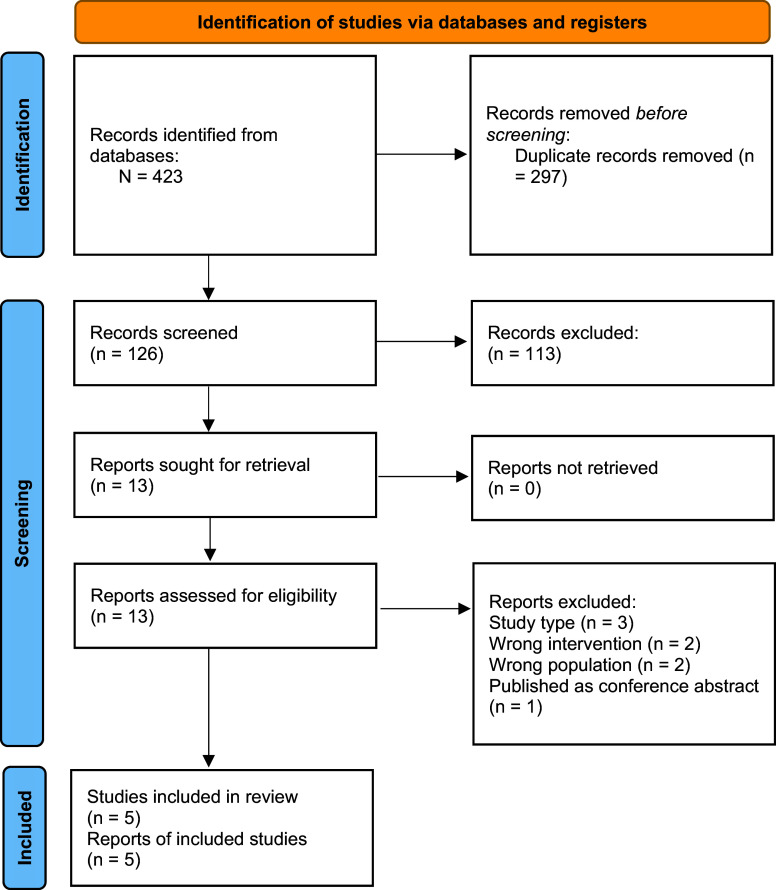
Flowchart for study selection.

After excluding duplicates, 126 records were screened. Of these, thirteen studies were selected for full-text reading, and five cost-effectiveness analyses were included (Figure 1).

The comparators adopted to repeat cytology were HPV testing, immediate colposcopy, and cytology (conventional or liquid-based) with reflex HPV testing. Three studies have two comparators (27–30), and one has three comparison groups (31).

The included studies (Table 2) were published between 2005 and 2016 in Brazil (28), Thailand (30), USA (27,31), and Sweden (29). Two evaluations are trial-based (27,29), one is based on retrospective cohort data (30), and the remainder follows a hypothetical cohort (28,31).

### Quality assessment

Based on the QHES tool, three studies were considered to have high quality (27–29), and two not high quality [(30,31); Table 1]. Common reasons for score downgrading were not presenting a clear declaration of the assumptions and a discussion of the magnitude and direction of potential bias.

**Table 1.** The main characteristics of the included studies

Author, year	Country of analysis	Analysis type	Tests	Decision model	Perspective	Time horizon	QHES
Ostenson, 2010	Sweden	Cost-effectiveness	Repeat cytology HPV testing Immediate colposcopy	Decision tree-based Markov model	Healthcare system	1 year	93
Kulasingam, 2006	USA	Cost-effectiveness	Repeat cytology HPV testing Immediate colposcopy	Decision tree	Payer	2 years	100
Hughes, 2005	USA	Cost-effectiveness	Conventional cytology + reflex HPV testing Liquid-based cytology + reflex HPV testing Repeat conventional cytology Immediate colposcopy	Not specified	Healthcare system	1 year	56
Tantitamit, 2015	Thailand	Cost-effectiveness	Repeat liquid-based cytology HPV testing Immediate colposcopy	Decision tree	Payer and patient	1 year	82
Vanni, 2010	Brazil	Cost-effectiveness	Repeat conventional cytology HPV test Immediate colposcopy	Markov	Healthcare system	Lifetime	100

Abbreviations: HPV, human papillomavirus

Hughes, 2005 (31) received the lowest score due to several limitations—no clear description of the methodology for data abstraction and estimation of resource quantities; insufficient time horizon; the model structure was not presented nor described, either the components of the numerator and denominator were displayed clearly; authors did not discuss the direction of potential bias; lack of sensitivity analysis; and finally, there was not a statement disclosing the funding source for study.

### Study perspective

Four studies adopted the healthcare system perspective (27–29,31), and one both the payer and the patient perspectives (30). Most of the studies that adopted the perspective of the healthcare system modeled public and private providers’ investment in triage programs, (Table 1). The payer perspective refers to a specific health provider in Thailand, the King Chulalongkorn Memorial Hospital (30).

### Decision model

Kulasingam, 2006 (27) and Tantitamit, 2015 (30) chose a decision tree, whereas Vanni, 2010 (28) chose a Markov model with a 6-month cycle length. Hughes, 2005 (31) did not specify the type of model.

Vanni, 2010 (28) adopted a lifetime horizon applying a five percent discount on costs and benefits yearly, Kulasingam, 2006 (27), a 2-year horizon, and the others a 1-year horizon (29–31).

All authors adopted modelling assumptions, as expected, but it was considered sufficiently described by Vanni, 2010 (28) and Kulasingam, 2006 (27). The most common health outcome was the number of CIN2+ cases detected. Other outcomes were the number of true positive results and life years saved (Table 3).

### Test characteristics and clinical parameters

The sensitivity and specificity values for the triage tests are shown in Table 2. When available, value ranges were also reported. Ostensson, 2010 (29) extracted data from a Swedish randomized clinical trial (32) and Kulasingam, 2006 (27) from the ALTS trial (33), and the detection rates measured were used to populate the model.

**Table 2.** Test performance estimates reported in the included studies

Study	Sensitivity	Specificity
Ostensson, 2010	HPV test (23–60 y): 0,82 HPV test (<30 y): 0,89 HPV test (>30 y): 0,75 Conventional cytology (23–60 y): 0,61 Conventional cytology (<30 y): 0,50 Conventional cytology (>30 y): 0,75	HPV test (23–60 y): 0,39 HPV test (<30 y): 0,22 HPV test (>30 y): 0,52 Conventional cytology (23–60 y): 0,56 Conventional cytology (<30 y): 0,52 Conventional cytology (>30 y): 0,59
Vanni, 2010	HPV test for CIN2: 0,94 (0,92–0,96) Colposcopy: 0,96 (0,95–0,97)	HPV test: 0,67 (0,58–0,76) Colposcopy: 0,48 (0,47–0,49)
Hughes, 2005	Conventional cytology: 0,62 (0,43–0,84) Liquid-base cytology: 0,81 (0,68–0,96) HPV test: 0,75 (0,33–1,0) Colposcopy and biopsy: 0,94 (0,81–1,0)	Conventional cytology: 0,89 (0,70–1,0) Liquid-base cytology: 0,78 (0,41–1,0) HPV test: 0,73 (0,51–0,95) Colposcopy and biopsy: 0,84 (0,52–1,0)

Abbreviations: CIN, cervical intraepithelial neoplasia; HPV, human papillomavirus

**Table 3.** Cost-effective strategy and incremental cost-effectiveness ratio reported in the included studies

Author, year	Outcome	Incremental cost-effectiveness ratio (USD 2024)	Cost-effective strategy
Ostensson, 2010	CIN2+ cases detected	385,66	Immediate colposcopy
Kulasingam, 2006	CIN3+ cases detected	1.768,17	HPV testing
Hughes, 2005	True positives	522,83	Liquid-based cytology with reflex HPV testing
Tantitamit, 2015 (21)	CIN2+ cases detected	83,00	Immediate colposcopy
Vanni, 2010 (24)	Years of life saved	2.809,23	HPV testing

Abbreviations: CIN, cervical intraepithelial neoplasia; HPV, human papillomavirus

Tantitamit, 2015 (30) extracted data from medical records of women assisted at the King Chulalongkorn Memorial Hospital and collected the detection rates to populate the model. Hughes, 2005 (31) employed various sources to extract main estimates and ranges for the different tests analyzed, mainly observational studies.

Vanni, 2010 (28) extracted data from two published meta-analyses. One regarding data for HPV testing (34) and the other for colposcopy data (35). Both meta-analyses provided aggregated accuracy ratios at a threshold of ASCUS or LSIL for an outcome of CIN2+ in women with abnormalities in cytology smears. Cytology accuracy was derived from an epidemiological mathematical model (36).

Clinical parameters included in the cost-effectiveness models were screening coverage, adherence to screening strategy, loss to follow-up, prevalence of low-grade and precursor lesions, HPV infection, and cervical cancer.

### Transition probabilities

Given the type of decision model adopted in each cost-effectiveness analysis, only Vanni, 2010 (28) applied transition probabilities to simulate the natural history of the lesions detected. They were derived from literature and whenever possible from studies conducted in Brazil or Latin America (37–39) as the target population of analysis was Brazilian women.

### Cost inputs

As no study adopted a societal perspective, only direct cost parameters were included in the cost-effectiveness analyses. In addition to the cost of test kits and analysis, the authors also considered the costs of appointments, follow-up exams, and treatment of CIN2+ and cervical cancer.

Kulasingam, 2006 (27) and Hughes, 2005 (31) retrieved unit costs from Medicare reimbursement values; Ostensson, 2010 gathered costs from three Sweden hospitals database: Stockholm South General Hospital, Danderyd Hospital, and Karolinska University (29); Tantitamit, 2015 (30) retrieved the main cost estimates from King Chulalongkorn Memorial Hospital, whereas the values for cost ranges in sensitivity analysis, were obtained from a survey of laboratories from various government and private hospitals in Thailand.

Vanni, 2010 (28) retrieved most costs from the Brazilian Private Healthcare System standard procedure table (Classificação Brasileira Hierarquizada de Procedimentos Médicos), except for medical visits, nurse visits, and day hospital, which were provided by the Hospital de Clínicas de Porto Alegre. The HPV testing had not been reimbursed by the health system at the time when the study was published, so the authors assumed the same cost as a cytology smear because similar or lower prices were achieved in other settings.

### Sensitivity analysis

Regarding sensitivity analysis, it was only not performed by Hughes, 2005 (31). Except for Vanni, 2010 (28), all the studies performed only one-way sensitivity analysis ranging the test accuracy parameters using the values of 95 percent confidence intervals from the literature estimates, and costs ranged on an arbitrary percentage.

Analyzing the difference in results with the variations, Kulasingam, 2006 (27) performed an interesting strategy to approximate the costs of all groups compared to the highest (immediate colposcopy) and lowest cost estimates (repeat conventional cytology).

Additionally, to one-way sensitivity analysis, Vanni, 2010 (28) performed probabilistic and threshold analysis.

### Study findings

In all cost-effectiveness analyses, repeat conventional cytology was less costly but less effective than comparators.

Comparing HPV testing and immediate colposcopy to repeat cytology, HPV triage presents a more cost-effective and equally or even more effective follow-up option for women aged 30 years or older. However, it may be dominated by immediate colposcopy in a further pairwise comparison depending on the unit cost of each test.

In Tantitamit, 2015 (30) for provider and patient perspectives, and in Ostensson, 2010 (29) for health system perspective, colposcopy was the most cost-effective strategy in comparison to HPV testing (Table 3).

Tantitamit, 2015 (30) sensitivity analysis, showed the most cost-effective strategy shifted away from colposcopy as the costs of colposcopy increased or the costs of cytology decreased. Lowering the costs of HPV testing would be necessary to enhance the cost-effectiveness of the follow-up strategy, as demonstrated in Ostensson, 2010 (29).

If liquid-based cytology were employed in primary screening, HPV triage would become significantly less expensive and more cost-effective compared to conventional cytology and immediate colposcopy.

When comparing liquid-based cytology to conventional cytology, there was an extra cost to detect each additional true positive result using the liquid-based method according to Hughes, 2005 (31). The immediate colposcopy strategy was both more expensive and less effective than liquid-based cytology with reflex HPV testing, thus being dominated by the latter. The study also demonstrated that ICER increased with an increase in patients’ age.

Kulasingam, 2006 (27) and Vanni, 2010 (28) found the HPV test to be the most cost-effective strategy compared to immediate colposcopy and repeat cytology. In Vanni, 2010, a threshold analysis study revealed that if the cost of HPV testing exceeded twice the cost of cytology, the optimal strategy would be immediate colposcopy and the discount rate did not change the conclusions of ICER magnitude (28).

## Discussion

This is the first systematic review aiming to investigate the cost-effectiveness of triage alternatives to replace repeat cytology in the management of women diagnosed with ASC-US and LSIL. This study fills a gap of robust economic evidence about this topic to be evaluated along with available clinical evidence in the updating of triage protocols.

In different contexts analyzed by five cost-effectiveness studies repeat conventional cytology was not the most efficient strategy. Strategies involving HPV testing as the main test, or as a reflex test was reported as cost-effective in three studies, whereas two studies found colposcopy to be more cost-effective.

Sensitivity analysis performed by all studies showed that ICER results were highly sensitive to variation in test unitary costs and accuracy estimates. Although a significant range in accuracy estimates can be observed through the studies due to the variability of literature sources employed in data extraction, the data follow the same tendency. Besides, these parameters were explored in sensitivity analysis in all studies, so it should not be considered a topic of high concern in future decision-making based on this systematic review.

The reason immediate colposcopy was cost-effective in Tantitamit, 2015 (30) and Ostensson, 2010 but not in Kulasingam, 2006 (27) and Vanni, 2010 (28), might be related to differences in some clinical and cost inputs included in the first two studies.

Tantitamit, 2015 (30) included loss to follow-up in HPV testing and cytology groups, with high rates of missed cases, but not in the immediate colposcopy group, which might have affected the effectiveness of HPV testing and cytology. Ostensson, 2010 (29) considered that after cytology and HPV testing, there would be a referral to gynecological consultation or a follow-up visit to a midwife, raising the follow-up costs in these groups, whereas the colposcopy group would not undergo these steps.

Although the preferred health outcome for economic evaluations is QALY (18,40), it was not evaluated by any of the five studies. Vanni et al., 2010 (28) justify that the choice of life years saved as the outcome was due to the absence of health utilities for Brazilian women eligible for triage or with cervical cancer in the literature.

Tantitamit, 2015 (30) also mentioned the decision for CIN2+ detection as an outcome of the lack of QALY data for the target population or a similar one. The detection of CIN2+, the main health outcome in the majority of studies included, has been considered a validated outcome to be used as a proxy for the occurrence of cervical cancer (41,42).

Subsequent investigations should be prioritized to obtain QALYs of the women who were diagnosed with ASC-US/LSIL smear results after performing different strategies to allow the comparability and interpretability of their findings regarding cost-effectiveness. Decision makers may encounter challenges in drawing decisive conclusions when confronted with a body of evidence characterized by diverse metrics of health benefits.

The comparison between the included studies was limited by the variability of the analyzed tests, decision models, health outcomes, resources, costs, and time horizon. In cost-effectiveness studies, a new intervention is usually compared to the standard practice, which differs according to the analyzed context. The ICER is strongly influenced by the comparator due to the nature of the calculation that includes the benefits and costs of the new intervention versus the comparator, and thus, the comparison of reported ICERs was challenging (43).

Given the nature of economic evaluations related to the analyzed topic, the choice of the best cervical cancer triage strategies is highly complex due to the number of criteria to consider from the perspective of health policies (21).

The included cost-effectiveness analyses were published at least a decade ago. Not only there is limited economic evidence on the ASC-US/LSIL population but also, the more recent studies are already evaluating the cost-effectiveness of more advanced strategies to overcome HPV testing shortcomings (16), emphasizing the urgent need for management updating in countries that still practice cytology-based triage.

In this sense, this systematic review can be useful by comparatively presenting parameters and assumptions of interventions, providing information on the application of decision analysis models, and guiding the health managers’ decisions on the implementation of new triage tests, considering the reality of each health system.

Thus, studies of this nature can contribute to other research and to actions aimed at expanding access to technologies and health services for the female population.

The simplification enabled by the alternative strategies in comparison to repeat cytology regarding the number of procedures required to complete triage, lower loss to follow-up, and increasing detection of true positives CIN2+, allows timely treatment that is turned into life years saved and efficient resource allocation.

## Supporting information

Meirelles et al. supplementary material Meirelles et al. supplementary material

## Supplementary material

The supplementary material for this article can be found at http://doi.org/10.1017/S0266462324000540.
