# Supplementary material for: Systematic review of economic evaluations of triage tests for women with atypical squamous cells of undetermined significance (ASC-US) or low-grade squamous intraepithelial lesions (LSIL)
Source: Int J Technol Assess Health Care. 2024 Nov 18;40(1):e58. doi: 10.1017/S0266462324000540 (PMC11579666; doi:10.1017/S0266462324000540)
Supplement: Meirelles et al. supplementary material [file S0266462324000540sup001.docx]

***Medline search strategy***

| Search | Strategy | Number of hits |
| --- | --- | --- |
| #1 | "Economics"[Mesh:NoExp] OR "Costs and Cost Analysis"[mh] OR "Economics, Nursing"[mh] OR "Economics, Medical"[mh] OR "Economics, Pharmaceutical"[mh] OR "Economics, Hospital"[mh] OR "Economics, Dental"[mh] OR "Fees and Charges"[mh] OR "Budgets"[mh] OR budget*[tiab] | 372,664 |
| #2 | economic*[tiab] OR cost[tiab] OR costs[tiab] OR costly[tiab] OR costing[tiab] OR price[tiab] OR prices[tiab] OR pricing[tiab] OR pharmacoeconomic*[tiab] OR "pharmaco-economic*"[tiab] OR expenditure[tiab] OR expenditures[tiab] OR expense[tiab] OR expenses[tiab] OR financial[tiab] OR finance[tiab] OR finances[tiab] OR financed[tiab] OR "value for money"[tiab] OR "monetary value*"[tiab] | 1,307,276 |
| #3 | "models, economic"[mh] OR "economic model*"[tiab] OR "markov chains"[mh] OR markov[tiab] OR "monte carlo method"[mh] OR "monte carlo"[tiab] OR "Decision Theory"[mh] OR "decision tree*"[tiab] OR "decision analy*"[tiab] OR "decision model*"[tiab] | 148,864 |
| #4 | #1 OR #2 OR #3 | 1,566,877 |
| #5 | "atypical squamous cells of the cervix"[MeSH Terms] OR ("atypical"[All Fields] AND "squamous"[All Fields] AND "cells"[All Fields] AND "cervix"[All Fields]) OR "atypical squamous cells of the cervix"[All Fields] OR ("atypical"[All Fields] AND "squamous"[All Fields] AND "cells"[All Fields] AND "undetermined"[All Fields] AND "significance"[All Fields]) OR "atypical squamous cells of undetermined significance"[All Fields] OR ("atypical squamous cells of the cervix"[MeSH Terms] OR ("atypical"[All Fields] AND "squamous"[All Fields] AND "cells"[All Fields] AND "cervix"[All Fields]) OR "atypical squamous cells of the cervix"[All Fields] OR "ascus"[All Fields]) OR ("Low-grade"[All Fields] AND "squamous"[All Fields] AND "intra-epithelial"[All Fields] AND ("lesion"[All Fields] OR "lesions"[All Fields] OR "lesional"[All Fields] OR "lesions"[All Fields])) OR ("squamous intraepithelial lesions"[MeSH Terms] OR ("squamous"[All Fields] AND "intraepithelial"[All Fields] AND "lesions"[All Fields]) OR "squamous intraepithelial lesions"[All Fields] OR "lsil"[All Fields]) | 9,171 |
| #6 | ((Triage[Title/Abstract] OR Early Diagnosis[MeSH Terms] OR Diagnosis, Early[Title/Abstract] OR Early Detection of Disease[Title/Abstract] OR Disease Early Detection[Title/Abstract] OR Early Detection of Cancer[Title/Abstract] OR Cancer Early Detection[Title/Abstract] OR Cancer Screening[Title/Abstract] OR Screening, Cancer[Title/Abstract] OR Cancer Screening Test*[Title/Abstract] OR Screening Test*, Cancer[Title/Abstract] OR Test*, Cancer Screening[Title/Abstract] OR Early Diagnosis of Cancer[Title/Abstract] OR Cancer Early Diagnosis[Title/Abstract]) AND (Mass Screening*[Title/Abstract] OR Screening*, Mass[Title/Abstract] OR Screening*[Title/Abstract])) | 62,638 |
| #7 | #5 AND #6 | 1,398 |
| #8 | #4 AND #7 | 151 |

***Embase search strategy***

| Search | Strategy | Number of hits |
| --- | --- | --- |
| #1 | (('economics'/de OR 'costs') AND 'cost benefit analysis'/exp OR 'pharmacoeconomics'/exp OR 'health economics'/exp OR 'fees') AND 'charges' OR 'budget'/exp OR 'budget*':ti,ab,kw OR 'economic*':ti,ab,kw OR 'cost':ti,ab,kw OR 'costs':ti,ab,kw OR 'costly':ti,ab,kw OR 'costing':ti,ab,kw OR 'price':ti,ab,kw OR 'prices':ti,ab,kw OR 'pricing':ti,ab,kw OR 'pharmacoeconomic*':ti,ab,kw OR 'pharmaco economic*':ti,ab,kw OR 'expenditure':ti,ab,kw OR 'expenditures':ti,ab,kw OR 'expense':ti,ab,kw OR 'expenses':ti,ab,kw OR 'financial':ti,ab,kw OR 'finance':ti,ab,kw OR 'finances':ti,ab,kw OR 'financed':ti,ab,kw OR 'value for money':ti,ab,kw OR 'monetary value*':ti,ab,kw | 1,716,675 |
| #2 | 'economic model'/exp OR 'economic model*':ti,ab,kw OR 'markov chain'/exp OR 'markov':ti,ab,kw OR 'monte carlo method'/exp OR 'monte carlo':ti,ab,kw OR 'decision theory'/exp OR 'decision tree*':ti,ab,kw OR 'decision analy*':ti,ab,kw OR 'decision model*':ti,ab,kw | 160,038 |
| #3 | #1 OR #2 | 1,831,283 |
| #4 | 'atypical' AND 'squamous' AND 'cells' AND 'undetermined' AND 'significance' OR 'atypical squamous cells of undetermined significance' OR 'atypical squamous cells of the cervix'/exp OR ('atypical' AND 'squamous' AND 'cells' AND 'cervix') OR 'atypical squamous cells of the cervix' OR 'ascus' OR ('low-grade' AND 'squamous' AND 'intra-epithelial' AND ('lesion' OR 'lesion s' OR 'lesional' OR 'lesions')) OR 'squamous cell lesion'/exp OR ('squamous' AND 'intraepithelial' AND 'lesions') OR 'squamous intraepithelial lesions' OR 'lsil' | 14,018 |
| #5 | ('triage':ti,ab,kw OR 'early diagnosis'/exp OR 'diagnosis early':ti,ab,kw OR 'early detection of disease':ti,ab,kw OR 'disease early detection':ti,ab,kw OR 'early detection of cancer':ti,ab,kw OR 'cancer early detection':ti,ab,kw OR 'cancer screening':ti,ab,kw OR 'screening cancer':ti,ab,kw OR 'cancer screening test*':ti,ab,kw OR (('diagnosis' OR 'screening' OR 'mass screening'/exp OR ('mass' AND 'screening') OR 'mass screening' OR 'early cancer diagnosis'/exp OR ('early' AND 'detection' AND 'cancer') OR 'early detection of cancer' OR 'screen' OR 'screenings' OR 'screened' OR 'screens') AND 'test cancer':ti,ab,kw) OR 'test cancer screening':ti,ab,kw OR 'early diagnosis of cancer':ti,ab,kw OR 'cancer early diagnosis':ti,ab,kw) AND ('mass screening*':ti,ab,kw OR 'screening mass':ti,ab,kw OR 'screening*':ti,ab,kw) | 86,919 |
| #6 | #4 AND #5 | 2,235 |
| #7 | #3 AND #6 | 264 |
